# Emergent Oscillations in Networks of Stochastic Spiking Neurons

**DOI:** 10.1371/journal.pone.0014804

**Published:** 2011-05-06

**Authors:** Edward Wallace, Marc Benayoun, Wim van Drongelen, Jack D. Cowan

**Affiliations:** 1 Department of Mathematics, University of Chicago, Chicago, Illinois, United States of America; 2 Department of Pediatrics, University of Chicago, Chicago, Illinois, United States of America; 3 Computation Institute, University of Chicago, Chicago, Illinois, United States of America; École Normale Supérieure, College de France, CNRS, France

## Abstract

Networks of neurons produce diverse patterns of oscillations, arising from the network's global properties, the propensity of individual neurons to oscillate, or a mixture of the two. Here we describe noisy limit cycles and quasi-cycles, two related mechanisms underlying emergent oscillations in neuronal networks whose individual components, stochastic spiking neurons, do not themselves oscillate. Both mechanisms are shown to produce gamma band oscillations at the population level while individual neurons fire at a rate much lower than the population frequency. Spike trains in a network undergoing noisy limit cycles display a preferred period which is not found in the case of quasi-cycles, due to the even faster decay of phase information in quasi-cycles. These oscillations persist in sparsely connected networks, and variation of the network's connectivity results in variation of the oscillation frequency. A network of such neurons behaves as a stochastic perturbation of the deterministic Wilson-Cowan equations, and the network undergoes noisy limit cycles or quasi-cycles depending on whether these have limit cycles or a weakly stable focus. These mechanisms provide a new perspective on the emergence of rhythmic firing in neural networks, showing the coexistence of population-level oscillations with very irregular individual spike trains in a simple and general framework.

## Introduction

Networks of the central nervous system display oscillations at many frequencies and scales of organization. Gamma oscillations (25–100 Hz) in cerebral cortex and hippocampus are implicated in a bewildering variety of neural phenomena, including many stages of sensory processing, and in a wide range of brain regions in many species [1]–[3]. Changes in the patterns of neuronal oscillations are linked to changes in brain states, such as attention and sleep-wake transitions, and to pathologies such as epilepsy and schizophrenia [2], [4], [5]. Mathematical models are crucial to understanding the mechanisms underlying the generation and function of these oscillations.

Models for oscillations in nervous tissue fall into three types, depending on whether the oscillations arise within the individual neurons and then synchronize across the network, emerge purely at the population level, or occur due to a combination of the two. The first case includes coupled oscillator models such as those involving simplified model neurons [6], or detailed models in parameter regimes where the individual neurons oscillate intrinsically, and the network oscillations arise from the synchronization of these individual oscillating elements [7]. The second case includes population-based models such as the Wilson-Cowan equations [8], which may display limit cycle oscillations in bulk variables which are coarse-grained representations of neuronal firing; however these may not be informative about how the spike times of individual neurons relate to the network oscillation. The third case includes the delay-driven models of Brunel et al [9], [10] and most models based on Hodgkin-Huxley neurons [11]–[13]. In both the first and third category we may have exact synchronous firing, where each neuron fires once per population cycle, or “cluster states” where neurons fire together in groups at some fixed multiple of the population frequency [14]. Noisy versions of such models may produce sparse or irregular firing, so that neurons skip beats, i.e. do not fire in every cycle of the network oscillation; but generally the spike times have a narrow distribution of phases within the network cycle.

In this paper we examine mechanisms by which oscillations emerge purely at the network level. We use a stochastic model of individual neurons which gives the elements no intrinsic oscillatory capacity but which makes the relationship of individual spike trains to the population oscillation transparent. The inspiration comes from complex systems beyond neural networks, where population-level oscillations without the individual components themselves oscillating are widespread. In ecology, oscillations occur in predator-prey systems in which the individual components are organisms, each of which may be born or die only once [15]; and in oscillating chemical systems such as the Belousov-Zhabotinsky reaction or the brusselator, molecules undergo reactions at effectively random times yet the overall concentrations fluctuate close to periodically [16], [17].

A stochastic network may oscillate when the mean-field equations follow a limit cycle, or also when the mean-field equations have a damped oscillation. In the latter case the noise causes a continual excitation which pushes the system away from its mean-field fixed point, causing a population-level resonant oscillation. This mechanism, called *quasi-cycles* [18], has been studied in ecology [19] and epidemiology [20].

There are two principal differences between the results presented here and those by Brunel and co-workers [9], [10] and Mattia and del Giudice [21]. Firstly, our oscillations are driven by excitatory-inhibitory feedback rather than by synaptic delay in inhibitory-inhibitory coupling, and so their frequency is strongly modulated by changing synaptic strengths or sparseness of connectivity, rather than determined primarily by a delay time. Secondly, individual neuron spike trains are far more irregular in our model, weakly rather than strongly biased towards peak phases of the population oscillation. The extreme irregularity of spike trains in our model is suggestive of the irregularity of spike trains in vivo. Additionally, our neurons are abstracted to 2-state Markov processes, which cannot oscillate individually, rather than integrate and fire neurons, which may have an internal resonance that gives rise to or interacts with population oscillations. We return to these differences, and discuss the key role played by noise, in the discussion.

We use the stochastic rate model [22], in which the simplified model neurons are 2-state random processes. Our earlier paper [22] demonstrated the existence of avalanche dynamics, irregular and aperiodic synchronous firing events, in some parameter regimes of the model; this paper addresses oscillations, which are periodic synchronous firing events, in different parameter regimes of the same model.

This paper begins by summarizing the stochastic rate model. We show that, if the “mean-field” equations of the network, which are the Wilson-Cowan equations, have a stable limit cycle oscillation, then the full network activity will be a noisy limit cycle. The population oscillation coexists with irregular spike trains whose multimodal inter-spike-interval distribution has its peaks at multiples of the oscillation period. We show that noisy limit cycle oscillations persist in sparse networks, whose frequency varies with parameters for synaptic weights and sparseness of connectivity as well as the single-neuron parameters. Then, we show quasi-cycle oscillations and calculate their frequency, which also depends on the connectivity parameters. We discuss the two mechanisms, comparing the individual activity with population-level behaviour, and noting the transition from one oscillatory regime to the other as parameters vary. Both mechanisms are characterized by a single major peak in the power spectrum and a roughly power law decay at high frequencies; the stochastic model is informative about the tail of the power spectrum, unlike a deterministic Wilson-Cowan model. In the discussion we address the relation to previous models in detail, and the biological implications of the work.

## Results

### Summary of the stochastic rate model

We begin by summarizing the stochastic rate model [22], presented in detail in the methods section. Individual neurons are approximated as coupled, continuous-time, two-state Markov processes. In this paper we model networks of 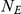 excitatory and 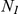 inhibitory neurons, initially with all-to-all connectivity, later extending to sparsely connected networks. At any given time, the state of a neuron is either active or quiescent. The decay rate, i.e. the transition rate from active to quiescent, is a constant 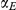 for active excitatory neurons and 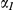 for active inhibitory neurons. The firing rate, i.e. the transition rate from quiescent to active, depends on the network state via the individual neuron's total input, 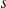. When the For quiescent excitatory neurons, the firing rate is
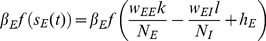
(1)and for quiescent inhibitory neurons
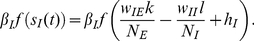
(2)Here 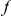 is a sigmoid *response function*,
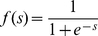
(3)giving the firing rate as a function of input, 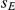 is the total synaptic input to excitatory neurons, consisting of the external input 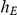, and internal terms involving synaptic weights 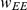 from excitatory to excitatory neurons, and so on. The choice of response function and the inclusion of population-dependent maximal firing rates 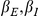 are the only differences in the model from [22].

The model specifies the rates of the transitions, but, to account for the presence of noise in actual biological networks, this is a stochastic process in which the time to the next event is a random variable; the network dynamics may be thought of as a random walk on a lattice, depicted in supplementary figure ??. We simulate the model according to Gillespie's stochastic simulation algorithm (see methods).

Our main analytical tool is the linear noise approximation, in which the number of neurons active in each population is approximated as the sum of a deterministic term, scaling with the population size, and a stochastic fluctuation term, scaling with the square root of this size:
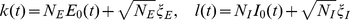
(4)The deterministic terms obey the exact Wilson-Cowan equations
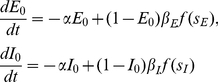
(5)where in terms of the new variables, the input currents are written
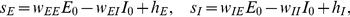
(6)The fluctuation variables 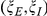 obey a linear stochastic differential equation
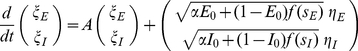
(7)where 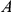 is related to the Jacobian matrix, or linearization, of (5), calculated at their (deterministic) solution, by a scaling transformation involving the population sizes. The derivation and details are summarized in the methods section.

### Population limit-cycle oscillations with weak single-neuron oscillations

**Figure 1:**
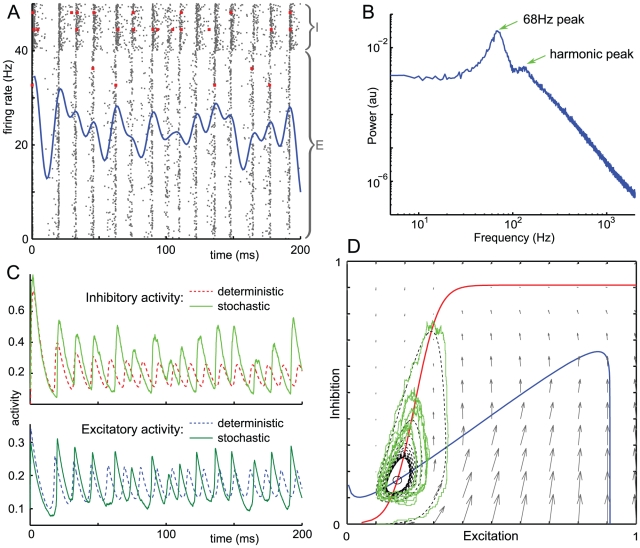
Noisy Limit Cycles. Simulations with parameter values 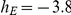, 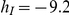, 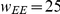, 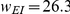, 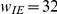, 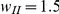, 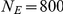, 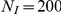, 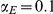, 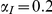, 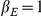, 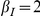. A: Mean firing rate of network (smoothed over 5 ms) plotted over raster plot of spikes (grey). Individual neurons are rows, with the 20% of inhibitory neurons plotted at the top, otherwise unsorted. Four individual spike trains are highlighted in red. The mean excitatory firing rate is 16.4 Hz and mean inhibitory firing rate is 45.2 Hz. B: Normalized power spectrum for the excitatory population in simulation showing peak at 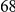 Hz. A diffuse subpeak around the 2nd harmonic of 136 Hz is also shown, followed by a power-law decay of 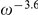 (linear fit to log-log plot from frequencies from 200 to 2000 Hz). C: Time series plot of the excitatory and inhibitory activity of trajectories from deterministic and stochastic models. The deterministic trajectories show a stable limit cycle with a period of roughly 11.3 ms, corresponding to an 89 Hz oscillation. D: Plot of phase plane of system, including the vector field (grey) and the 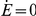 (blue) and 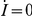 (red) nullclines of the deterministic Wilson-Cowan equations. Sample trajectories of the deterministic (black dashed) and stochastic (light green) system are shown.

First we describe population-level oscillations in the stochastic rate model for a set of parameter values where the deterministic Wilson-Cowan equations produce limit cycles. As the raster plot in figure 1A shows, in this scenario neurons tend to fire in periodic bursts. The power spectrum of the network's excitatory activity (figure 1B) shows a peak at 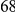
Hz, in the gamma band, a harmonic subpeak at twice that frequency, and at frequencies up to 2000 Hz, shows a 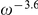 decay. Despite the high frequency network oscillation, individual excitatory neurons fire with a mean rate of 16.4 Hz and inhibitory neurons with a mean rate of 45.2 Hz. In fact, no neuron in the network fires more than 61 times in any given second of the simulation, which is less than the frequency of the network oscillation. This phenomenon is termed cycle-skipping [23], since individual neurons do not fire with every peak in the network oscillation. Stochastic cycle-skipping is not surprising in our model since the individual neurons have no intrinsic oscillatory capability when isolated from the network. The noisy limit cycle oscillations are an emergent property of the interaction of neurons at the network level.

Figures 1C and 1D compare time series of the stochastic model with the deterministic Wilson-Cowan equations. The deterministic system exhibits stable limit-cycle oscillations with a frequency of 89 Hz, while the stochastic trajectory exhibits undamped oscillations at a lower frequency band centered on 68 Hz. The discrepancies between these frequencies arises from the interaction of noise and nonlinearity in the stochastic system, the noise arising from random spike times. After an initial transient, the stochastic network follows an irregular pattern of spontaneous activity near, but usually outside, the deterministic limit cycle.

Noisy perturbations to the trajectory may be decomposed into a component transverse to the limit cycle and another parallel to it. Perturbations transverse to the limit cycle will cause fluctuations in the amplitude of the excitatory activity. Since the limit cycle is stable, these transverse fluctuations are damped over time. However, fluctuations away from the centre of the limit cycle are more persistent since they are amplified by the larger vector field in this region of the phase plane. Thus the stochastic system tends to take longer and slower loops surrounding the limit cycle; this accounts for the frequency of the noisy system being lower than that of the deterministic system. Unlike the transverse direction, there is no restorative force for perturbations in the parallel direction, so perturbations that change the phase of the oscillation accumulate over time until the relative phase of the deterministic and stochastic trajectories become independent, a phenomenon known as phase slipping [24].

**Figure 2:**
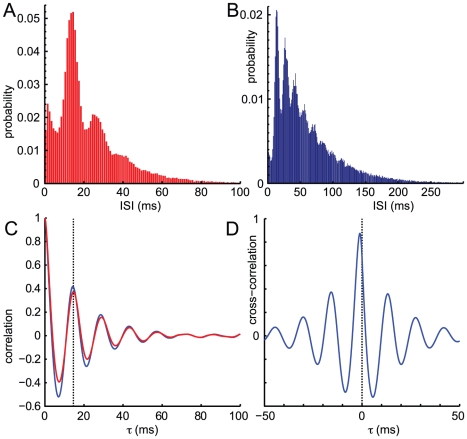
Inter-spike intervals and cross-correlations for noisy limit cycles. Results from a 10-second simulation using parameters from figure 1. A: Inter-spike interval (ISI) histogram for the inhibitory population (131,435 data points) B: ISI histogram for the excitatory population (89,290 data points). C: Normalized autocovariance (AC_0_F) for inhibitory (red) and excitatory (blue) activity, showing a peak in the inhibitory AC_0_F at 14.9 ms corresponding to the oscillation period. D: Cross-correlation of excitatory and inhibitory activity, showing that the excitatory phase leads the inhibitory phase by 1.1 ms.

Every individual neuron fires irregularly, sometimes skipping an oscillation cycle, sometimes firing once or even twice within one oscillation period. This may be seen from the two inhibitory and two excitatory spike trains highlighted in the raster plot of figure 1A. The inter-spike interval (ISI) histogram for the inhibitory population in figure 2A clarifies this, showing a small peak at 2 ms indicating that some neurons fire twice in the same cycle, a large peak at 14 ms representing spikes separated by one cycle, a smaller peak at 28 ms corresponding to spikes separated by two cycles, and a slow decay with 19% of ISIs longer than two cycle periods. The excitatory population has a lower firing rate so we expect its ISIs to be more dispersed: the excitatory ISI histogram in figure 2B shows a small peak at 2 ms indicating that some neurons fire twice in the same cycle, a large peak at 14 ms representing spikes separated by one cycle, then 5 discernible subharmonic peaks and a slow decay in which 82% are separated by more than one cycle. This indicates that often neurons cycle-skip, i.e. do not fire in two consecutive cycles. The presence of peaks at several integer multiples of the oscillation period indicates that the firing is not clustered into several groups of neurons each firing at a fixed multiple of the population period. Since the ISI histogram has no empty bins, spikes may occur at any phase of the population oscillation, although some phases are much less likely than others.

The presence of some very short ISIs is possible in our model because there is no absolute refractory period, so that a neuron may fire a spike, then transition back to the quiescent state, and then fire another spike, in an arbitrarily short amount of time. Although this is possible, it is highly improbable and we feel that the small proportion of unphysiologically short ISIs is a harmless artefact.

The normalized autocovariances (AC_0_F) of population activity in figure 2C indicate oscillations preserving phase information for the first 2-4 cycle periods but an almost complete loss of phase information over the duration of 6 cycle periods. The first peak in the inhibitory AC_0_F is at 14.9 ms (black dotted line), and in the excitatory AC_0_F at 14.3 ms, corresponding to frequencies of 69.9 Hz and 67.1 Hz respectively, consistent with the power spectrum peak at 68 Hz in figure 1B. The cross-correlation of excitatory and inhibitory activity in figure 2D shows that the oscillation involves excitatory-inhibitory feedback, where the excitatory firing tends to lead the inhibitory firing by 1.1 ms. The cross-correlation has a decay of phase information comparable to that of the autocovariance.

### Noisy limit cycles persist in sparse networks

**Figure 3:**
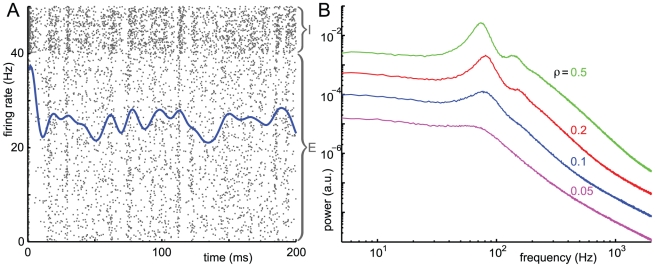
Noisy limit cycles persist in sparse networks. Simulations of random sparse networks with parameter values 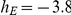, 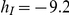, 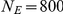, 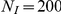, 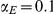, 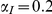, 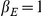, 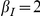. For a given connectivity, individual nonzero synaptic weights are scaled to give the Wilson-Cowan weight parameters 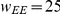, 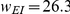, 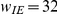, 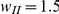, see text for details. A: For network with 10% connectivity (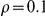), mean firing rate of network (smoothed over 5 ms) plotted over raster plot of spikes (grey). Individual neurons are rows, with the 20% of inhibitory neurons plotted at the top, otherwise unsorted. The mean excitatory firing rate is 16.2 Hz and mean inhibitory firing rate is 45.2 Hz B: Excitatory power spectra for different connectivity levels, 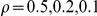 and 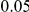. The top trace is normalized to 1 and subsequent traces are normalized to 0.1, 0.01, and 0.001, respectively, i.e. displaced downwards 1, 2 or 3 units in log co-ordinates.

Population-level oscillations may be produced in sparse networks by the same mechanisms at work in all-to-all connected networks. Figure 3A shows oscillations in a network with 1000 neurons and random homogenous 10% connectivity. Weakly synchronous firing is detectable from the vertical stripiness of the raster plot; the power spectrum (blue trace in figure 3B) has a diffuse peak at 75 Hz. By “random homogenous connectivity,” we mean the synaptic connection from any neuron to any other is nonzero with probability 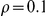 independently of other connections, as in an Erdös-Rényi random graph. However, the strength of the nonzero synapses varies with population so that the Wilson-Cowan approximation is identical to the limit cycles simulation in the all-to-all case in the previous section. To see how this is achieved, consider the synaptic connections from inhibitory to excitatory neurons. The term 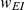 in the Wilson-Cowan equations represents the mean connection strength to the excitatory population from the inhibitory population, multiplying the proportion of inhibitory neurons active, 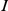. The mean input 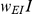 thus depends on the strength of individual synapses 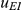, and on the number of synaptic inputs, which is on average a product of the number of inhibitory neurons 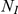 and the density of connections 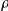:
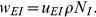
(8)Analogous formulas hold for the other pairs of populations.

As the network becomes increasingly sparse, the frequency of population oscillations changes while their amplitude declines, shown in Figure 3B. At 50% connectivity the power spectrum has a peak at 73 Hz and a subpeak at the 2nd harmonic. At 20% connectivity the peak is at 81 Hz, is smaller in magnitude and the harmonic subpeak is much smaller. At 10% connectivity the peak is at 76 Hz and considerably more diffuse, while the harmonic subpeak is absent. At 5% connectivity there is no peak, rather the power spectrum stays close to flat until roughly 70 Hz before beginning a faster decay.

Figure 3B also illustrates how a stochastic network becomes increasingly unlike its approximating Wilson-Cowan equations as the connectivity declines. Randomness in an all-to-all network's dynamics arise only from the random spike times of individual neurons, but once a neuron has fired the whole network responds with an identical change in firing probabilities. In a sparse network, any two neurons receive inputs from different groups of neurons, so that when a neuron fires only its postsynaptic neighbours change their firing probabilities. The inputs of neurons in one population are correlated, but not identical, random variables, so that the transition rates are heterogenous, depending not just on the numbers of neurons active per population but on the particular combination of neurons active. The random connection probabilities act as a second source of noise in the network, which grows as the sparseness increases; indeed, if there are 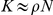 incoming synapses to a neuron, a Gaussian approximation of input will have variance scaling with 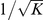 rather than the network size parameter 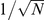. The Wilson-Cowan approximation ignores the extra variance and the input correlations within the network since it relies upon making a population average over the synaptic inputs, 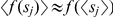. This explains why the sparser the connectivity, the less the network dynamics resemble the Wilson-Cowan approximation.

#### Frequency in sparse networks varies consistently with Wilson-Cowan equations

We have shown that the stochastic model undergoes noisy limit cycles when the Wilson-Cowan equations predict limit cycles; however, the frequency of the noisy limit cycles is somewhat different from the frequency of the deterministic limit cycle. This raises the question, how good a guide is the deterministic system to the stochastic system? In particular, does the frequency of noisy limit cycles vary with parameters in a manner consistent with that predicted by the deterministic system?

**Figure 4:**
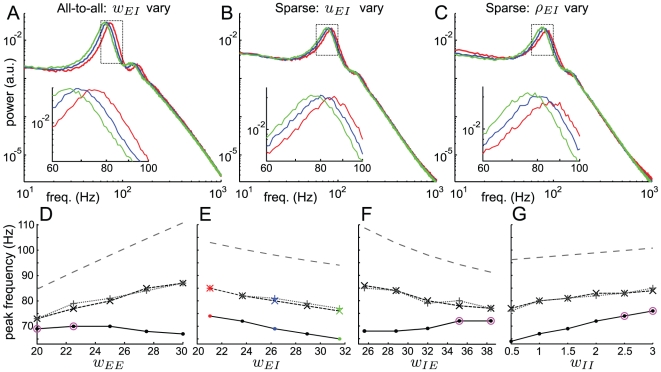
Oscillation frequency in the stochastic model varies with Wilson-Cowan parameters. A-C: Power spectra for networks with 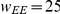, 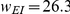, 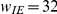, 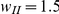; insets show the peaks of those power spectra. Synaptic weights are scaled to give the Wilson-Cowan weight parameters 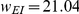 (red), 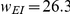 (blue) or 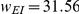 (green), with others fixed at 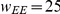, 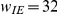, 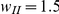, see text for details. A: Varying synaptic strength in all-to-all network. B: Varying synaptic strength 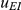 in sparse network with density of connections 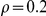. C: Varying inhibitory-to-excitatory connection density 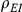 in sparse network with other connection densities 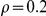. D-G: Oscillation frequencies for networks varying one parameter from 80%-120% of original value (see methods for details). Solid line with 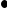, vary synaptic strength in all-to-all network; small dashed line with 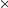, vary synaptic strength in sparse network; grey dotted line with 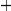, vary connectivity in sparse network; grey large dashed line, frequency from imaginary part of eigenvalue at fixed point of deterministic system. Magenta circles denote parameter values for which the deterministic system has a stable fixed point, so that the stochastic system displays quasi-cycles rather than noisy limit cycles. All simulations in this figure have parameter values 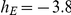, 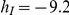, 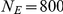, 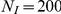, 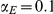, 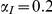, 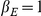, 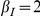.

Figure 4 shows that the answer is a qualified yes. Figure 4A shows that, in the all-to-all network with parameters as in figure 1, increasing the inhibitory-to excitatory synaptic input 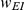 causes the power spectrum peak to move to the left. Figure 4E compares the peak frequency (solid line) with that predicted by the eigenvalues at the fixed point of the Wilson-Cowan equations (grey dashed line), which shows the same downwards trend. This means that the network oscillation frequency decreases as predicted by the deterministic equations.

In a sparse random network, one may vary either a synaptic strength or a sparseness parameter in order to change a single connectivity parameter in the Wilson-Cowan equations. Similarly to the previous section, the term 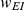 in the Wilson-Cowan equations represents the mean connection strength to the excitatory population from the inhibitory population. Here we allow the density of connections to vary on a population-by-population basis, so that 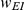 depends on the strength of individual synapses 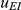, and on the number of synaptic inputs, which is on average a product of the number of inhibitory neurons 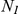 and the density of inhibitory-excitatory connections 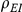:
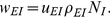
(9)Analogous formulas hold for the other pairs of populations. Figure 4 shows that in a sparse network with 20% connectivity, increasing either the relevant synaptic strength 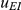 (figure 4B) or the connection density 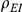 (figure 4C) causes the power spectrum peak to move to the left; meanwhile the tail of the power spectrum varies very little. This means that the network oscillation frequency decreases as 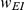 increases. Tracking only the peaks of these power spectra in figure 4E is consistent with this. As we move the parameters corresponding to 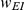 between 80% and 120% of their original value, the system displays noisy limit cycles with a consistently decreasing oscillation frequency. The grey dashed line in figure 4E shows the frequency predicted from the imaginary part of the eigenvalue at the fixed point of the Wilson-Cowan equations, although as we discuss the deterministic limit cycle frequency is different from this. The plot shows that the varying the parameters of the stochastic network corresponding to 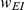 has an effect on the frequency corresponding to that predicted by the eigenvalue of the deterministic system.

Figures 4D, 4F and 4G show further that varying the parameters of the sparse network corresponding to 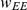, 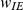 and 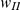 has a similar effect on the frequency corresponding to that predicted by eigenvalues of the deterministic system. However, the all-to-all network shows a more complex effect; in most but not all cases the variation in frequency corresponds. The points marked by magenta circles in figure 4 are those for which the related deterministic system has a stable fixed point. We think that the discrepancy is related to the underlying Hopf bifurcation and return to this point in the discussion, after presenting results on quasi-cycles in the next section.

### Population quasi-cycle oscillations without single-neuron oscillations

Here we examine quasi-cycles, a form of oscillatory activity that can only exist in stochastic systems. The deterministic simplification of the system is characterized by a stable focus associated with damped oscillations; the stochastic component perturbs this equilibrium and causes sustained oscillations. Quasi-cycles were noticed independently in a similar neural network model by [25], where the single-neuron spike trains were not addressed.

**Figure 5:**
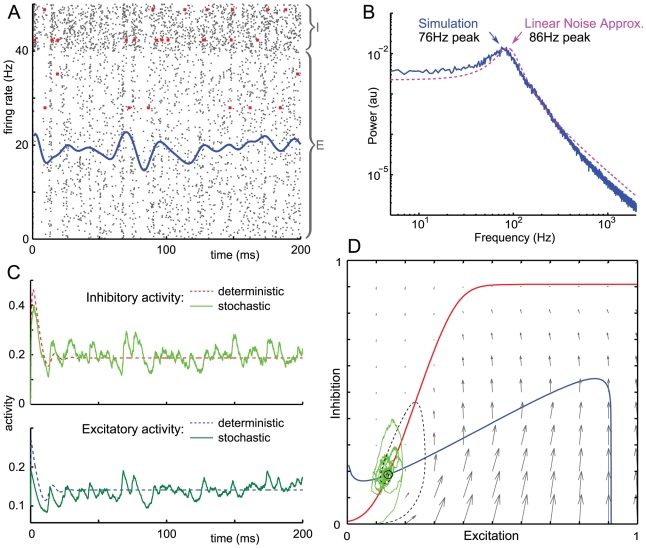
Quasi-cycles in an all-to-all network. Simulations with parameter values 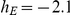, 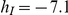, 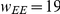, 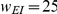, 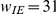, 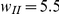, 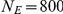, 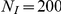, 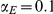, 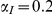, 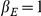, 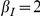. A: Mean firing rate of network (smoothed over 5 ms) plotted over raster plot of spikes (grey). Individual neurons are rows, with the 20% of inhibitory neurons plotted at the top, otherwise unsorted. Four individual spike trains are highlighted in red. The mean excitatory firing rate is 14.1 Hz and mean inhibitory firing rate is 39.2 Hz B: Normalized power spectrum for the excitatory population in simulation (blue) with peak at 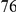
Hz and from the linear noise approximation, 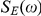 (magenta, see text), with peak at 86 Hz. The simulation shows a roughly 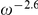 decay at frequencies up to 2000 Hz. C: Time series plot of the excitatory and inhibitory activity of trajectories from deterministic and stochastic models. D: Plot of phase plane of system, including the vector field (grey) and the 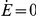 (blue) and 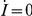 (red) nullclines of the deterministic Wilson-Cowan equations. Sample trajectories of the deterministic (black dashed) and stochastic (light green) system are shown.

The network tends to fire in periodic bursts, as the raster plot in figure 5A shows. The power spectrum of the excitatory activity (figure 5B) shows a diffuse peak at 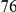
Hz, in the gamma band, and at higher frequencies, shows an 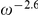 decay. Despite the high frequency network oscillation, individual excitatory neurons fire with a mean rate of 14.1 Hz and inhibitory neurons with a mean rate of 39.2 Hz. In fact, no neuron in the network fires more than 58 times in any given second of the simulation, which is less than the frequency of the network oscillation. Stochastic cycle-skipping is not surprising since the individual neurons have no intrinsic oscillatory capability when isolated from the network. Quasi-cycle oscillations are an emergent property of the interaction of neurons at the network level.

Figures 5C and 5D compare time-series of the stochastic model to the deterministic Wilson-Cowan equations with. The deterministic system exhibits damped oscillations with a frequency of 86 Hz about a stable fixed point, while the stochastic trajectory exhibits undamped oscillations at a slightly lower frequency band centered on 76 Hz. The effect of random spike times of individual neurons is to add noise to the dynamics. In portions of the phase plane where the Wilson-Cowan equations predict fast deterministic dynamics, the effect of noise is small by comparison; near the fixed point of the deterministic system, where the Wilson-Cowan equations predict slower dynamics, the effect of noise is proportionately larger. After an initial transient, the stochastic network follows an irregular pattern of spontaneous activity where noise pushes the system away from the deterministic fixed point enough to induce oscillations with a predictable frequency.

#### Quasi-cycle oscillations are explained by a Gaussian approximation

The fluctuating terms are close to those predicted by the linear noise approximation in equation (27). Since the Wilson-Cowan system here has a single attractive fixed point, after an initial transient the Jacobian matrix 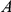 and noise amplitudes in (27) approach a constant.

Taking the Fourier transform of (27) allows us to approximate the power spectrum of the fluctuations, giving the squared amplitude of the oscillations in each frequency band. A standard calculation, summarized in the appendix, shows that the power spectrum for the excitatory activity consists of a delta peak at zero and a fluctuating component
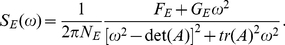
(10)where 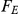 and 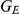 are calculated from the coefficients of (27), themselves calculated at the deterministic solution 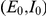. This power spectrum has a peak at 85.7 Hz, (rounded to 86 Hz in figure 5B), slightly below the minimum of the denominator 
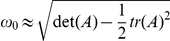
, and decays proportional to 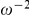 for very large frequencies. The power spectrum of inhibitory activity has analogous behaviour, with a peak at 89.1 Hz, also close to 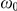.

We can now ask, for what size network the deterministic Wilson-Cowan equation is a good approximation to the full stochastic system? For the simulation shown in figure 5 the calculated amplitude of the Fourier component at its maximum of 85.7 Hz is
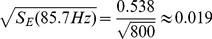
(11)which is within an order of magnitude of the deterministic solution, 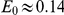. Similarly
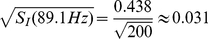
(12)is comparable to the deterministic solution 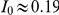. Despite the large factors of 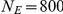 or 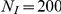 in the denominator of (10), the term 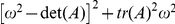 is small enough at the peak frequency that the power at this frequency is large. Note that the peak 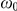 may be close to the value that minimizes the denominator of (10), so that simply minimizing the denominator gives an approximation to the peak frequency. However, the quadratic term in the numerator ensures that minimizer is not exactly the peak frequency.

In the simulation presented here, there is a 10 Hz difference between the peak frequency in the simulation and that predicted by the linear noise approximation. The curves match better in networks with less noise or further from the Hopf bifurcation (data not shown).

#### Population oscillations are undetectable from individual spike trains

**Figure 6:**
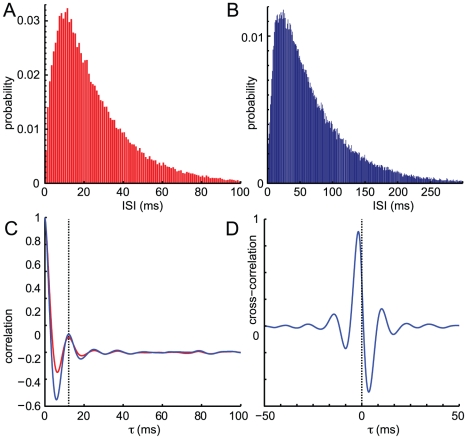
Inter-spike intervals and cross-correlations for quasi-cycles. Results from a 10-second simulation using parameters from figure 5. A: Inter-spike interval (ISI) histogram for the inhibitory population (111,879 data points) B: ISI histogram for the excitatory population (78,028 data points). C: Normalized autocovariance (AC_0_F) for inhibitory (red) and excitatory (blue) activity, showing a peak in the inhibitory AC_0_F at 12.2 ms corresponding to the oscillation period. D: Cross-correlation of excitatory and inhibitory activity, showing that the excitatory phase leads the inhibitory phase by 1.7 ms.

Every individual neuron fires irregularly, sometimes skipping several oscillation cycles, sometimes firing more than once within an oscillation period, as can be seen in the two inhibitory and two excitatory spike trains highlighted in the raster plot of figure 1A. The inter-spike interval (ISI) histograms in figure 6 clarifies that population quasi-cycles are not detectable in the spike trains of individual neurons. The inhibitory ISI histogram in figure 6A shows a single large peak at 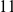
ms and a slow decay. The excitatory ISI histogram in figure 6B similarly has a single peak and slow decay, although it is more dispersed since the excitatory firing rate is much lower than the inhibitory firing rate. This indicates that single neurons cannot be meaningfully said to oscillate in quasi-cycle dynamics, and that the oscillations are only detectable at the population level.

The autocorrelation functions (AC_0_F) in figure 6C indicate oscillations with an almost complete loss of phase information over 2-3 periods. This is a much quicker loss of phase information than in the limit cycle case, a point we return to in the discussion. The peak in both AC_0_Fs, at 12.2 ms (black dotted line), corresponds to a frequency of 82 Hz, close to the power spectrum peak at 76 Hz in figure 5B. The cross-correlation of excitatory and inhibitory activity in figure 6D shows that the oscillation involves excitatory-inhibitory feedback, where the excitatory firing tends to lead the inhibitory firing by 1.7 ms. The cross-correlation has a decay of phase information comparable to that in figure 2C.

## Discussion

Our results show two forms of oscillations in the stochastic rate model of neural activity: noisy limit cycles, and quasi-cycles. Both of these involve a single peak on the power spectrum of network activity indicating population-level oscillations (figures 1B and 5B). yet irregular firing of individual neurons (figures 1A and 5A). The power spectrum shows a slow decay after its peak similar to experimental measurements. These oscillatory dynamics are robust to variations in connectivity over a range of at least 80%–120% of their original values (figure 4), and to changes in the density of connections from 100% to 10% connectivity (figure 3). The firing of individual neurons has an even weaker phase relationship with a quasi-cycle population oscillation than to a noisy limit cycle oscillation, to the extent where the population oscillation is undetectable from the spike trains of single neurons in the network.

Our analysis treated the stochastic model as a noisy perturbation of the deterministic Wilson-Cowan equations [8]. Noisy limit cycles occur when the deterministic Wilson-Cowan equations have a stable limit cycle. The stochastic system then undergoes what we call noisy limit cycles, which are oscillations localized near the deterministic limit cycle and so of similar frequency (figure 1C–D). We simulated networks where the connectivity is made increasingly sparse, which makes the network's evolution increasingly unlike that predicted by the deterministic equations. In this case, the oscillations persist while becoming more irregular, down to connection probabilities as low as 10% in a network of 1000 neurons (figure 3). Although the deterministic system only approximates the frequency of noisy limit cycle oscillations, in sparse networks, the oscillation frequency varies with parameters in a manner largely consistent with the deterministic Wilson-Cowan equations (figure 4). Quasi-cycles occur when the deterministic Wilson-Cowan equations have a stable fixed point approached via weakly damped oscillations; then the stochastic model undergoes continued fluctuations localized in frequency near that of the damped oscillations (figure 5). This is because the noise acts as a source of excitation, pushing the system into the oscillatory neighbourhood of its fixed point. In the case of quasicycles, we calculated the power spectrum of the network activity explicitly and showed its dependence on network parameters. Analytical results approximated the frequency of the oscillation and the shape of the power spectrum (figure 4B).

Both these forms of synchronous fluctuations differ from the neuronal avalanches we reported in a previous paper [22]. We do not consider the avalanches to be oscillations as there is no peak in the power spectrum, and so no preferred frequency. Avalanches, noisy limit cycles, and quasi-cycles are distinct forms of synchronous firing grouped into network bursts that emerge from excitatory-inhibitory interactions in noisy networks, but avalanches are aperiodic while noisy limit cycles and quasi-cycles are oscillatory. Neuronal avalanches and quasi-cycles both arise from stochastic destabilization of a stable fixed point, in the avalanche case from a stable node via functionally feedforward connectivity, and in the quasi-cycles case from a stable focus generating weakly damped oscillations.

Exploring the links between these dynamical regimes would shed light on the emergence of oscillations from avalanches reported in cell cultures by Gireesh & Plenz [26]. In particular, it suggests that during cortical development, network parameters may change in such a way that the underlying Wilson-Cowan approximation changes from functionally feedforward to weakly damped oscillations; this transition deserves further study.

### Distinguishing different mechanisms driving oscillations

How should we distinguish data from noisy limit cycles and quasi-cycles, given that the power spectra of both mechanisms are characterized by a single peak and power-law decay decay at higher frequencies? The limit cycle power spectrum in figure 1B has a smaller peak around the 2nd harmonic which is not discernible in the quasi-cycle power spectrum in figure 5B. However, the appearance of harmonic peaks in limit cycle oscillations is sensitive to the shape of the limit cycle, for example, a circular limit cycle traversed at constant speed would not have harmonic peaks. The harmonic peak also disappears in sparsely connected networks undergoing noisy limit cycles (figure 3). Comparing the tails of the power spectra far above the peak frequency, the limit cycle power spectrum exhibits power law decay with a higher exponent than that of quasi-cycles.

The autocovariance of activity was used to distinguish limit cycles and quasi-cycles in ecological models by Pineda-Krch et al [27], since limit cycles preserve phase information better than quasi-cycles. The autocovariance is sensitive to the mean activity as well as amplitude of oscillations, so here we use a related metric, the normalized autocorrelation function (AC_0_F). Figures 2C and 6C depict the AC_0_F; comparison of the results reveals much lower amplitude AC_0_F oscillations for quasi-cycles which nearly vanish within a few periods, while the AC_0_F oscillations for noisy limit cycles are larger in amplitude and are maintained for many periods, indicating a longer phase memory in the limit cycle case. Pineda-Krch et al. [27] suggested that the amplitude of the oscillation at a lag time of the period of the network oscilation could be used to differentiate the two dynamics, offering a “heuristic threshold. ” Since the threshold they offered, like the metric they used, is sensitive to the relative size of the mean activity and the amplitude of the population oscillations, we do not recommend it. However, the relative decay of the AC_0_F generally exhibits much slower decay in its oscillations for noisy limit cycles than for quasi-cycles.

Figure 6A–B show the precision in phase memory from the perspective of individual neurons in the network by examining their inter-spike intervals (ISI). For quasi-cycles, there is a single peak in the ISI histogram for both excitatory and inhibitory neurons, roughly corresponding to the period of the network oscillation, followed by monotonic decay in the ISI. The exponential-like tail of the ISI at time scales above the oscillation period means that population oscillations are undetectable from the spike trains of individual neurons. By contrast, the ISI distributions of figures 2A–B describe a different situation where individual neurons are more tightly linked to the network oscillation. There are peaks in the ISI corresponding to firing once every three and even four cycles of the network oscillation. The ISI distribution decays monotonically on time scales much larger than 60 msec.

Another finding in the limit cycle case is another peak in the inhibitory ISI distribution at 2 msec (figure 2A), related to the model's lack of absolute refractory period. This suggests that our model neurons often fire in bursts with 2 msec delays during a single peak of the network oscillation, contrasting with the main peak ISI of 14 ms msec corresponding to the oscillation period.

The two forms of population oscillations also respond differently to changes in the noise amplitude. We have conceptualized the stochastic network's dynamics as a small stochastic perturbation of a deterministic system, expressed in equations (25) and (27). Noisy limit cycles were viewed as a perturbation of a deterministic limit cycle, and quasi-cycles as a perturbation of a deterministic stable focus. So for noisy limit cycles, the deterministic term oscillates and the stochastic term perturbs these oscillations, whereas for quasi-cycles the deterministic term goes to a stable fixed point while oscillatory fluctuations persist in the stochastic term. This means that, as the noise amplitude decreases, we expect a noisy limit cycle to continue oscillating with increasing regularity in amplitude and phase, whereas quasi-cycle oscillations, which are noise-driven, would decrease in amplitude and remain irregular. This is experimentally testable in nervous tissue by injecting white noise current, in the first case weakening population oscillations and in the second case enhancing them.

In our model, due to the scaling of synaptic strength with population size 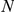, the noise amplitude varies roughly as 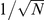, and so noisy limit cycles would be enhanced and quasi-cycles attenuated as the network size increases. However, the relationship of synaptic strength to network size *in vivo* is not well understood.

Despite our conceptualization of the system's dynamics as a small stochastic perturbation of a deterministic system, both the deterministic and stochastic population variables arise from exactly the same microscopic stochastic dynamics. In the stochastic rate model there is no clean separation between trend and noise; nor is there a clean separation between one deterministic regime and another. This is seen here most clearly in figure 4, where the peak of the power spectrum moves continuously as the deterministic system goes through a Hopf bifurcation from limit cycle to stable focus. Raster plots, power spectra, ISIs, and other statistics also vary slowly (data not shown); there is no discontinuity in the stochastic system corresponding to the bifurcation in the deterministic system. Thus we are forced to see noisy limit cycles and quasi-cycles as dynamical regimes which, although they are sometimes distinguishable, are not separated by a clear boundary [28]. This is a particular case of the general phenomenon that noise acts to blur the boundaries between dynamics which would be qualitatively different in a purely deterministic system [29].

In the light of this we return to the question of what causes the discrepancies between frequency trends with parameters in the different plots in figures 4D and 4F. For these parameter ranges, the deterministic limit cycle surrounds an unstable fixed point, and the noisy limit cycle explores the neighbourhood of the deterministic cycle. The eigenvalue at the fixed point (grey dashed trace in figures 4D–G) gives the frequency of the limit cycle born when it first emerges at a Hopf bifurcation, but becomes a less accurate approximation as the limit cycle moves further away from the fixed point. The limit cycle frequency could either increase or decrease relative to the eigenvalue's predictions, depending on both the length of the limit cycle trajectory and on the speed or strength of vector field along that trajectory.

In noisy networks trajectories may explore the exterior of the limit cycle, so that the frequency of the noisy system is lower than that of the deterministic system. A stochastic trajectory may also explore the interior, including the vicinity of fixed point, causing phase slips and resembling more a quasi-cycle system. In other words, in a noisy nonlinear system there are many competing effects as parameters vary and it is hard to predict which will dominate. The larger the noise amplitude, the less the stochastic system will resemble the deterministic system; and in the sparse case the fixed randomness in the weights acts as a second source of noise. This extra noisiness in sparse networks means that noisy limit cycle trajectories explore a larger region of the phase plane, including the interior of the limit cycle, meaning that the period of the oscillations will be derived from averaging the dynamical behaviour over this entire region, including the fixed point. We are not aware of perturbative approaches which are usefully predictive in these very noisy situations, but thankfully it is possible to explore the full stochastic system directly with simulations.

### Relation to experimental findings

Noisy limit cycles and quasi-cycles are mechanisms of emergent oscillations in neural networks, both of which allow the maximal firing rates of individual neurons to be lower than the network oscillation frequency. This phenomenon, referred to as cycle skipping, has been observed during physiological oscillations in visual cortex and during pathological oscillations in the form of fast ripples [23], [30], [31]. This suggests that the mechanisms presented here are good candidate mechanisms for such neuronal oscillations with cycle-skipping.

Power law tails in the power spectrum of neuronal networks are widely observed in vivo from electrocorticogram [32], [33] and electroencephalogram recordings [34]. These power laws naturally emerge in noisy limit cycles and quasi-cycles, or indeed in the stochastic rate model in other dynamical regimes [22]. In general, stochastic models for oscillations provide an entire power spectrum, both the peak and tail of which may be compared with experimental observations. We should note the distinction between the power law approximation, taken by fitting a portion of the power spectrum in physiologically measurable frequencies such as the 200–2000 Hz in figures 1B and 5B or 80–500 Hz in [33], and the asymptotic behaviour of an analytic expression such as (10) as 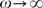, which may be approached at frequencies too high to be physiologically relevant. Only the former may be meaningfully compared with experimental results.

Differences in spike-time reliability between quasicycles and limit cycles should also be detectable experimentally. This may be the mechanism behind the observation that low frequency oscillations (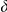
- and 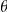
- band) exhibit weaker spike-time reliability than higher frequency oscillations (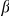
- or 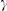
- band), or that the fast ripples seen pre-ictally are more disorganized than lower frequency ripples found in human hippocampus [5], [35]. This network mechanism accounting for the characteristics of oscillations at different frequencies is an alternative to mechanisms involving single-cell dynamics exhibiting some kind of resonance.

Figure 4 shows that the oscillation frequency in sparse networks varies with either a network-level parameter, the connectivity between a pair of populations, or with a single-neuron parameter, the synaptic strength between a pair of populations. This suggests a homeostatic mechanism controlling the frequency of network oscillations: changes in the pattern of connectivity could be compensated for by changes in synaptic strengths, or vice versa, meaning that the network frequency could be robust to changes induced by plasticity. It would also be interesting to investigate how connectivities more structured than the random graphs investigated here, or structured distributions of non-zero synaptic weights, affect a sparse network's oscillatory behaviour, and to compare the results with experiments. Biological networks are almost never all-to-all connected, and since the stochastic rate model is adaptable to sparse networks, it appears to be a useful tool for investigating these questions.

### Relation to other models

Studies by Brunel and co-workers, summarized in [10], and other groups [21], examine similar “sparsely synchronized oscillations” in networks consisting of leaky integrate-and-fire neurons with injected noise and delays in synaptic transmission. A single inhibitory population produces a delay-induced limit cycle whose frequency is strongly tied to the delay time; excitatory neurons modulate the frequency of this oscillation via interpopulation feedback. This is a distinct mechanism from those presented in this paper. Our model has no delays; moreover in the parameter sets presented here the inhibitory-inhibitory feedback is more than an order of magnitude smaller than the other connectivity parameters, whereas in the models of Brunel et al. the inhibitory-inhibitory feedback with delay drives the population oscillations. The results in figure 4 confirm that our noisy limit cycle oscillations are due to excitatory-inhibitory feedback. Moreover, comparison of the raster plots in figures 1 and 5 with the firing-rate plots in, for example, [9], shows that in Brunel et al.'s model the inhibitory neurons have a more definite phase relationship with the population oscillation. The extreme irregularity of spike trains in our model is suggestive of the irregularity of spike trains in vivo, and suggests that our model applies to a different set of experimental data.

In addition, the simplified model neurons used here provide for easier analysis and comparison with mean-field models such as the Wilson-Cowan equations; by contrast, Brunel et al. use integrate and fire neurons which account for more biophysical features of neurons.

Beyond the weak relationship of neuronal firing to the the phase of the population oscillation, the decay of the AC_0_F in the noisy limit cycle case means that phase is not very clearly defined in the case of noisy limit cycles; there is certainly no asymptotic phase one could use to establish isochrons. The work of Boland et al. [28] defines the phase of a point on the stochastic trajectory as the phase of nearest location to it on the deterministic trajectory. However, it is not clear how one would define phase from noisy limit cycles observed in data where a deterministic limit cycle is not analytically presented. In the case of quasi-cycles the trajectory frequently approaches the phase singularity at the fixed point, and the AC_0_F decays very quickly, suggesting that the idea of phase may not be useful in this context.

Delays in synaptic transmission are present in actual neural networks, so one wants to investigate what effect such delays would have on population oscillations in the present model. Because the time to spike firing is a random variable, there is already a random delay before a change in input triggers a spike. Consequently we would expect transmission delays much shorter than the typical inter-spike interval to have a negligible effect on the system's dynamics beyond slowing the oscillation frequency.

As neurons in larger networks are more likely to be far apart, we might expect conduction delays to play a bigger role in larger, spatially distributed networks. Longer delays could have major effects, including introducing another mode of population oscillations similar to that of Brunel et al. [10], which could interact with the present mechanism in non-obvious ways. Extending the current model to incorporate delays could be relatively straightforward to simulate as an adaptation of the Gillespie algorithm to account for delays already exists [36], and related algorithms are also able to incorporate delays [37].

We discussed the relationship of the stochastic rate model to other neural models in a previous paper [22], noting that discrete-state Markov models have been used to model neural dynamics at different timescales, for example up and down states in cortex in studies of repeating patterns of activity by Roxin et al [38], where state transitions operate 2 to 3 orders of magnitude slower than the present model. This raises the possibility that oscillations might arise from the same mechanisms, of noisy limit cycles or quasi-cycles, in a much lower frequency band such as the delta band (1–4 Hz). Indeed, there is no reason that the mechanisms presented here should be restricted to gamma-band oscillations; any other part of the nervous system with a stable focus or limit cycle in its dynamics, and noise, could produce emergent oscillations, at a frequency given by its own network characteristics.

The present work contributes to a wider body of literature addressing the ways in which noise contributes to biological network dynamics. Noise may work to create new dynamical behaviours such as avalanches [22]. Noise may also extend the parameter regimes for behaviours such as stochastic synchronization in feedforward networks [39], contrasting with the recurrent network presented here. On a smaller scale, noise may destabilize the fixed point of an excitable single neuron, causing oscillations whose period is linked to the time taken for an escape trajectory to return to the fixed point, a phenomenon called coherence resonance [40]. This contrasts with quasi-cycles, in which noise causes a network near a stable focus to explore the oscillatory neighbourhood of its fixed point, or noisy limit cycles, which are a perturbation of a deterministic limit cycle. Since simple stochastic models may produce phenomena only seen in deterministic models with more complex interaction terms, an overly deterministic viewpoint may encourage needless complexity in biological models [15], [41].

### Conclusions

The population oscillations presented here, coexisting with irregular single-neuron spike trains, arise from the interplay of noise and nonlinearity. Our stochastic excitatory-inhibitory network is essentially two-dimensional, and a deterministic system in two dimensions has a limited range of possible dynamics. A solely deterministic model with a stable sink will only be able to produce damped oscillations, and a deterministic model with a stable limit cycle will only produce perfectly periodic population firing, which is rarely if ever found in biological networks. Incorporating noise introduces a richer dynamical repertoire, including noisy limit cycles and quasi-cycles, without introducing extra variables or more complex microscopic dynamics. Moreover, stochastic models of oscillations produce an entire power spectrum, not merely the peak frequency, further enabling comparison with experimental results.

We have presented two related mechanisms for the generation of spontaneous population oscillations in nervous tissue; here we speculate on their functional role. Firstly, a change in the external input from one fixed level to another could move the network into or out of a state where it spontaneously produces noisy limit cycles or quasi cycles, analogously to a similar change in external input moving a deterministic system through a bifurcation. Secondly, here we have shown that for a wide range of parameters and connectivities, a stochastic network may spontaneously produce oscillations, so long as the mean values of input and synaptic strengths are within appropriate ranges. This raises the possibility that some kind of structure encoded into the choice of nonzero synaptic strengths could coexist with the generation of spontafoneous oscillations. We would then expect that, for different structured external inputs with similar means, a network might produce population oscillations with the same frequency, but with different groups of neurons firing at the peak of the oscillations. This potential mechanism for turning a rate code into a discrete code, phase-locked to spontaneous population oscillations, deserves further study.

## Methods

### The stochastic rate model

The stochastic rate model [22] treats neurons as coupled, continuous-time, two-state Markov processes. Each neuron can exist in either the *active* state 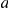, representing a neuron firing an action potential and its accompanying refractory period, or a *quiescent* state 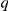, representing a neuron at rest. The transition probability for the 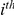 neuron to decay from active to quiescent is
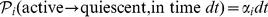
(13)as 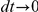, where 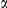 represents the decay rate of the active state of the neuron. The transition probability for the 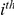 neuron to spike, i.e. to change from quiescent to active, is
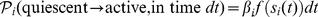
(14)as 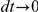. Here 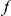 is a sigmoid *response function*,
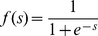
(15)giving the firing rate as a function of input, with maximum rate 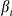, and 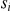 is the total synaptic input to neuron 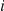. This total input is the sum of two terms,
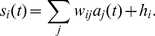
(16)The network input is 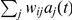, where 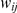 are the weights of the synapses, and the activity variable 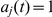 if the 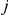
th neuron is active at time 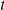 and zero otherwise. The term in 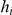 represents the net difference of external input to and threshold or bias of the 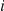
th neuron; in this study the origins of the net difference are unimportant, and we shall not address the question of time-varying external input. We use the Gillespie algorithm [42], an event-driven method of exact simulation, for all simulations of the master equation (see methods).

If a neuron receives constant input 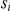, its inter-spike-interval is the sum of two independent exponential random variables, with parameters 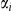 and 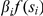 respectively, so its spike train will be irregular. In other words, these model neurons have no intrinsic capacity to oscillate.

Although there is no explicit refractory state in the model, in all simulations, 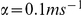, corresponding to an active state with a time constant of 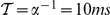 (1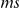 for the action potential plus 9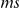 to approximate a refractory period where neurons are hyperpolarized). This choice of 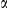 constrains neuronal firing rates to be no greater than 100 Hz.

### Network setup and the linear noise approximation

We next consider networks of 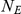 excitatory and 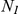 inhibitory neurons, initially with all-to-all connectivity depending only on the cell type; later in the results section we address how our findings extend to sparse connectivities. The outgoing synaptic weight from each excitatory neuron to each excitatory neuron is 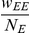, from inhibitory to excitatory is 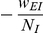, from excitatory to inhibitory is 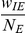, and from inhibitory to inhibitory is 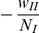.

The network's stochastic evolution can be thought of as a random walk between states with 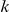 excitatory and 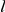 inhibitory neurons active, where the number of active neurons can increase or decrease only by one at a time, causing the state to wander around on a lattice as shown in Figure S1.We have summarized the input currents as
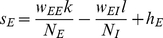
(17)

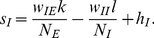
(18)


It is possible to write down a master equation for the network [22], [43], which would contain exactly the same information as Figure S1,and about which a limited amount may be analytically determined. That master equation, describing the evolution of the probabilities 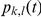 that the network is in state 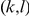 at time 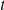, is
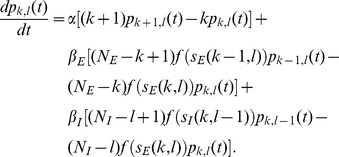
(19)Its derivation is presented in detail in [22], and the only difference here is in the inclusion of population-dependent maximal firing rates 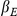 and 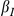, and a different choice of sigmoid response function (see equation 3). The analogous equation presented in [44] assumes that the proportion of active neurons is very small, and so lacks the saturation factors 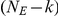 and so on; and also treats synaptic weights on a per-neuron rather than the per-population basis found here, accounting for the lack of a factor of 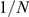 in their input currents.

### The linear noise approximation

Here we move to a tractable approximation called the linear noise approximation.

Suppose there is a timescale 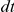 at which the numbers of spike and decay transitions in each population are large but the transition rates do not change appreciably; this occurs roughly when 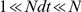. Since the rates do not change appreciably, the individual transitions are approximately independent, so the totals approximate Poisson random variables with mean equal to the product of the number of neurons in a state, the time step 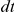, and the transition rate. Since the numbers of transitions are large, we may approximate each Poisson increment by a normal random variable (denoted 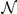) with identical mean and variance. The evolution of 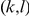 is then described by
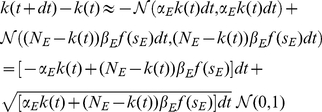
(20)Similarly, the inhibitory population increments are approximated as
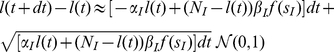
(21)Changing variables to the excitatory activity, 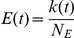 and the inhibitory activity 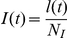, and dividing equations (20) and (21) by 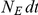 and 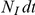 respectively, we arrive at
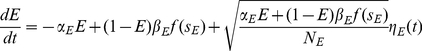
(22)

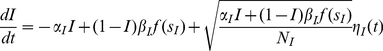
(23)where 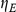 and 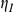 are independent white noise variables. These are nonlinear nonautonomous Langevin equations analogous to the chemical Langevin equation of Gillespie [45].

As the population sizes 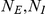 become very large, the noise terms scaled with 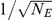 or 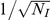 become proportionately smaller and equations (22) approach the deterministic Wilson-Cowan equations [8]. If 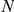 is quite large but stochastic effects are still important, we may make a further Gaussian approximation, representing the activity 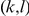 as the sum of a deterministic component 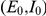 scaled by the population sizes, and a stochastic perturbation 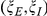 scaled by square root of the population sizes, so that
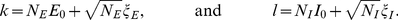
(24)Then the deterministic terms obey the exact Wilson-Cowan equations
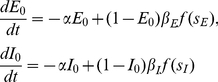
(25)where in terms of the new variables, the input currents are written
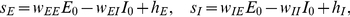
(26)The fluctuation variables 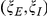 obey a linear stochastic differential equation
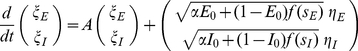
(27)where 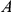 is related to the Jacobian matrix, or linearization, of (25), calculated at their (deterministic) solution, by a scaling transformation involving the population sizes, detailed in the following section. Equations (25) and (27) describe the *linear noise approximation*; one may measure the quality of this approximation by re-deriving it as a truncation of an infinite-order expansion using the small parameters 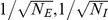, discussed in [22], [24], [46], [47].

Let us clarify the relationship between *activity* and *firing rate* in this model. Excitatory activity 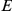 is the proportion of neurons currently active, and so the excitatory firing rate is 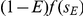 Hz per neuron. Conversely, if in the 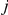
th timebin, of (small) width 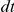, there are 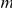 excitatory spikes and activity is initially 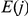, then the expected activity in the next timebin is
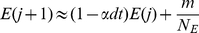
(28)where the first term represents remaining active neurons from the previous timestep and the second term the proportion of neurons which became active due to spiking at that timestep.

### Calculating coefficients of the linear noise approximation

Starting with equations (22–23), we use the expressions
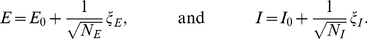
(29)to expand the equations (22–23) as a Taylor series about the deterministic terms 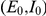. The zeroth-order terms are the deterministic Wilson-Cowan equations in (25). The perturbation terms then obey the equations
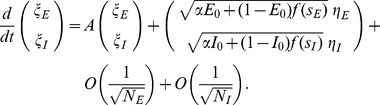
(30)The matrix for the linearized part is
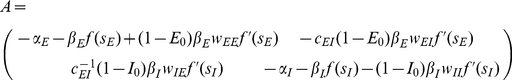
(31)where 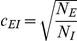. Varying the ratio 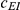 does not alter the determinant or trace of 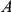, and so has no effect on its eigenvalues; if 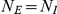 then 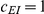, so 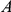 simplifies to the Jacobian of the deterministic system.

If the deterministic system is at a fixed point, then
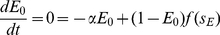
(32)

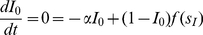
(33)so that the noise amplitudes multiplying the white noise (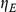 and 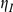) terms in (30) simplify to 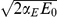 and 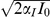 respectively.

### Simulation method

We simulate the entire network as a single continuous-time Markov process, using Gillespie's exact stochastic simulation algorithm [42]. The most general form of this starts with the single-neuron transition rates, that for the 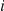
th neuron being:
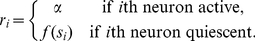
(34)The algorithm takes the state of the network, i.e. each neuron is specified as being either active or quiescent, and proceeds as:

1. Find neuronal transition rates 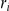, and network transition rate 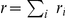.
2. Pick time increment 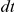 from an exponential distribution of rate 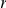.
3. Pick 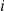
th neuron with probability 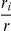, change its state, and update time to 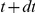.

In the case of homogenous all-to-all networks, if one only wants to simulate the number of neurons active in each population, one may simplify this algorithm along the lines of Gillespie's original presentation for a well-mixed chemical system, since the upwards transition rates 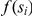 would be identical for all neurons in a population. The simplified algorithm uses much less memory and runs considerably faster.

All simulations were performed in Matlab 7.1 (Mathworks, Natick, MA); code is available on the corresponding author's website.

### Temporal coarse-graining

To produce plots of the mean firing rate, we counted the number of spikes 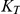 in timebins of width 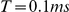, and convolved with a Gaussian of width 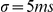. Some figures show an approximation to the proportion active: since active neurons decay at rate 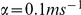, we may calculate the activity from the spike times as 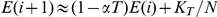.

### Time-averaged normalized power spectrum

The activity signal 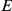 calculated using the temporal coarse-graining method described above. This signal 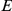 was then demeaned, removes any DC offset arising from the deterministic solution and scaling the total power to unity. In order to calculate the average power spectrum of 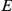, we divided the normalized 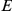 into 100 epochs (in figures 1 and 5) or 1000 epochs, each one second in duration, calculated the power spectrum of each epoch, and took the mean of these spectra. This reduced the noise in the overall power spectrum, and ensured resolution in increments of 1 Hz. To find the peak location we further smoothed the power spectrum with a 5-point triangular window, and then reported the frequency, necessarily a whole number of Hz, at which the curve was maximized. To estimate the exponent of the tail of the power spectrum, we transformed the data into log-log co-ordinates and then a least-squares linear fit to the frequencies from 200 to 2000 Hz.

### Calculating the power spectrum analytically from the linear noise approximation

Here we review how to calculate the power spectrum from a stable linear stochastic differential equation such as (30). This calculation is standard, and presented in, for example, [48]. The general vector form of such equations is
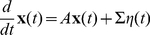
(35)which may be Fourier transformed to
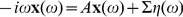
(36)so that
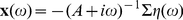
(37)Now, taking expectations we get the power spectrum
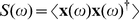
(38)

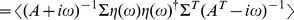
(39)

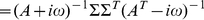
(40)where superscript 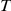 denotes the transpose and 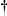 the conjugate transpose.

We have a two-dimensional linear system (30) governing the fluctuations, 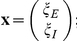 the matrix 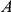 will be labelled as
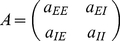
(41)whose components are detailed in (31). The noise amplitude matrix is given by
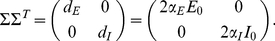
(42)


Then, the power spectrum in (40) has diagonal components
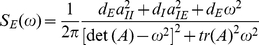
(43)

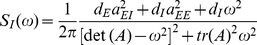
(44)which can be calculated numerically once the fixed point is determined. Note the form of the denominator. If 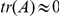, then the power spectrum indicates a resonance at 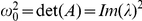. In other words, there is a peak in the power spectrum near the frequency of the damped oscillation in the deterministic Wilson Cowan system. Near 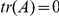, there will be a peak in the power spectrum at
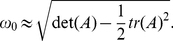
(45)Equations (43) and (44) also show the 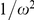 decay at higher frequencies.

Since 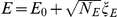, the power spectrum of excitatory activity 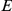 is the sum of a delta-peak at zero and the spectrum of 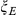 from (43) scaled by 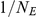; and likewise for the inhibitory activity.

### Inter-Spike Intervals

To make the ISI histograms, we extracted spike trains of individual neurons from the simulations, and stored all the ISIs. Since all neurons in the excitatory population are statistically identical, we then took the histogram of ISIs from all excitatory neurons together, and likewise for the inhibitory neurons.

### Autocovariance

We used Matlab's xcov function to calculate the autocovariance (ACF) of the excitatory and inhibitory population activity respectively, and then divided this by the variance to obtain the normalized autocovariance (AC_0_F). For the cross-correlation of excitatory and inhibitory activity, we analogously divided the output of xcov by the product of the standard deviations of each activity.

## Supporting Information

Figure S1Two-population network dynamics visualized. If there are *k* excitatory and *l* inhibitory neurons active, another excitatory neuron may become active, and network state moves rightwards one spot, at net rate 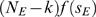, where 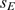 is the total synaptic input to an excitatory neuron. The rates for other transitions out of the state (*k, l*) are shown with black arrows and discussed in the population dynamics section of the results. Grey arrows represent transitions into the state (*k, l*) from adjacent states.(EPS)
